# TLR-2 Signaling Promotes IL-17A Production in CD4^+^CD25^+^Foxp3^+^ Regulatory Cells during Oropharyngeal Candidiasis

**DOI:** 10.3390/pathogens4010090

**Published:** 2015-03-17

**Authors:** Natarajan Bhaskaran, Samuel Cohen, Yifan Zhang, Aaron Weinberg, Pushpa Pandiyan

**Affiliations:** Department of Biological Sciences, School of Dental Medicine, Casfe Western Reserve University, Cleveland, OH 44106, USA; E-Mails: nxb160@case.edu (N.B.); shc30@case.edu (S.C.); ycz@case.edu (Y.Z.); axw47@case.edu (A.W.)

**Keywords:** T_reg_, Foxp3, Th17, IL-17A, TLR ligands, TLR-2, *Candida albicans*, IBD

## Abstract

Recent studies show that CD4^+^CD25^+^Foxp3^+^ regulatory cells (T_regs_) produce effector cytokines under inflammatory conditions. However, the direct role of microbial agents that serve as toll-like receptor (TLR) ligands in the induction of effector cytokines in T_regs_ is less clear. Here we show that CD4^+^Foxp3^+^T_regs_ produce the effector cytokine IL-17A during oropharyngeal candidiasis (OPC) and inflammatory bowel disease in a TLR-2/Myd88 signaling dependent manner. TLR-2 ligands promote proliferation in T_regs_ in the presence and absence of TCR signals and inflammatory cytokines *in vitro*. The proliferation is directly dependent on TLR-2 expression in T_regs_*.* Consistent with this, *Tlr2*^−/−^ mice harbor fewer thymically derived T_regs_ and peripheral T_regs_ under homeostatic conditions *in vivo*. However, under Th17 inducing conditions, IL-6 and TLR-2 signaling both in T_regs_ as well as antigen presenting cells (APC) are critical for maximal ROR-γt and IL-17A up-regulation in Foxp3^+^ T_regs_*.* The minimal and transient loss of Foxp3 expression and suppressive properties are due to the presence of IL-6 in the milieu, but not the direct effect of TLR-2 signaling in T_regs_. Taken together, our data reveal that TLR-2 signaling promotes not only proliferation, but also IL-17A in T_regs_, depending on the cytokine milieu. These IL-17A producing T_regs_ may be relevant in mucosal infections and inflammation.

## 1. Introduction

T_regs_ play important roles in dominant tolerance and immune homeostasis [1,2], and are targeted for human immunotherapy. Therefore, it is essential to study how T_regs_ precisely respond under steady state conditions and inflammatory conditions. Although T_regs_ are conventionally non-producers of inflammatory cytokines, recent reports document the presence of interleukin (IL)-17+Foxp3+ cells in mucosal environments in mice as well as in several human diseases [3,4,5,6,7]. This novel subset of cells likely represents an intermediate differentiation stage between Th17 cells and T_regs_. However, the mechanism by which they are induced, and their exact functions remain unknown [6,8]. TLR-2 recognizes bacterial lipopeptides, lipoteichoic acid from Gram-positive bacteria, zymosan from yeast cell walls, and the synthetic lipoprotein Pam3CSK4 [9]. Toll like receptors (TLR)s are generally expressed by the innate immune cells and APC, and play critical roles in host defense [10,11]. T lymphocytes, including T_reg_ cells demonstrate Toll-like receptor signaling as well [12]. TLR-2 polymorphisms are associated with changes in neonatal T_reg_ numbers as well as allergies and atopic dermatitis [13]. *In vitro*, Pam3CSK4 directly acts on CD4 cells and enhance their IL-17 production during Th17 differentiation [14]. However, research on TLR-2 signaling in T_regs_ has produced some varied results. Some studies show that TLR-2 ligands reduce Foxp3 expression in T_regs_, and reverse their suppressive functions [15,16]. Others show that TLR-2 ligands improve the survival of T_regs_ without reversing their suppressive functions [17]. By inducing peripheral T_regs_ (pT_regs_), TLR-2 signaling also promotes gut tolerance and not inflammation [18]. Though some of the above studies imply that suppression by T_regs_ may be overcome by TLR ligand induced DC maturation, and pro-inflammatory cytokine induction in effector cells [19,20], whether some of the effects were directly mediated by TLR-2 signaling in T_regs_, remains incompletely defined. Additionally, some data suggest that T_regs_ may play a deleterious role during acute disseminated candidiasis [21], however we as well as others have previously shown their protective functions during acute OPC and reinfection [22,23,24]. Whether T_regs_ themselves produce effector cytokines was not investigated at the time. In this study, we have shown that during OPC and under Th17 inducing conditions *in vitro*, TLR-2 ligands promoted proliferation and IL-17A induction in Foxp3^+^T_regs_. Increased proliferation was dependent on TLR-2 expression in T_regs_. However, IL-17A production was dependent on TLR-2 signaling in T_regs_ and APC, as well as IL-6 in the milieu. Under Th17 inducing conditions*,* though the presence of IL-6 in the milieu minimally reduced Foxp3 expression, TLR-2 ligand stimulation did not directly reduce Foxp3 expression. The TLR-2 activated T_regs_, which included IL-17 producing T_regs_, retained suppressive activity. Taken together, we have identified the direct role of TLR-2 ligands in promoting proliferation and IL-17A production in T_regs_*,* without affecting their suppressive functions *in vitro* and *in vivo.*

## 2. Results

### 2.1. CD4^+^CD25^+^Foxp3^+^ T_regs_ Express IL-17A During Oral *C. Albicans* Infection and Inflammatory Bowel Disease (IBD) *in Vivo*

**Figure 1:**
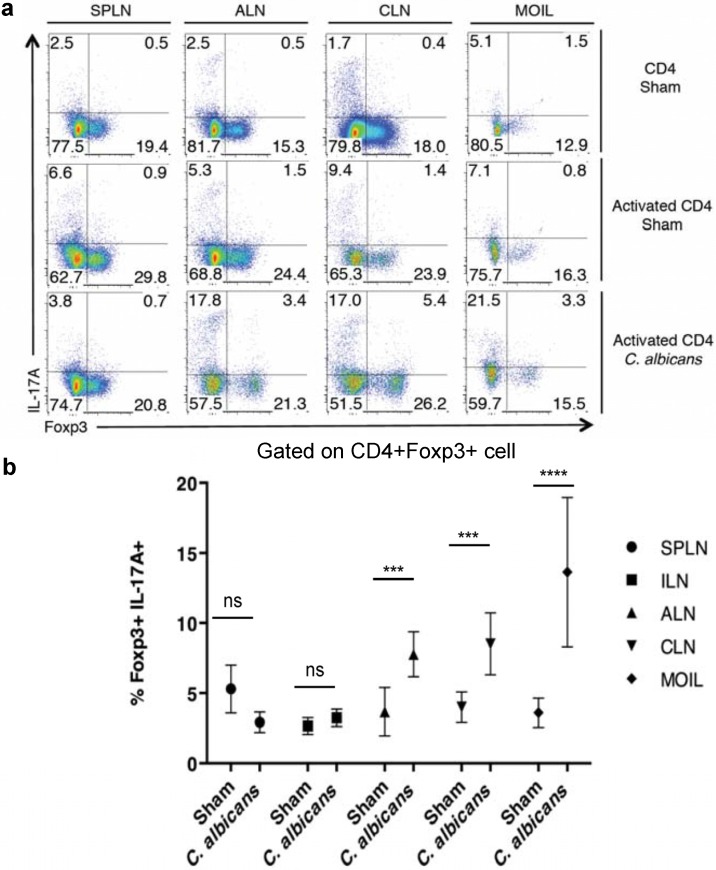
CD4^+^Foxp3^+^ T_regs_ express IL-17A during *C. albicans* infection *in vivo*. (**a**) Foxp3^GFP^ reporter mice were infected with PBS sham or *C. albicans*. On day 2 after infection, the cells from spleen, lymph nodes, and MOIL were isolated and restimulated for cytokine analyses. Flow cytometric plots showing Foxp3 and IL-17A expression, gated on CD4+ cells (top), and CD4+CD44^high^ activated CD4 cells (middle and bottom rows). (**b**) Cells were isolated as in “a”. Flow cytometric analyses of IL-17A expression, gating on CD4+Foxp3+ cells in sham or *C. albicans* infected mice. Data represent triplicate experiments.

We and others have previously shown that IL-17A is critical for anti-Candidal host resistance [22,25], and T_regs_ enhance acute effector Th17 responses and fungal clearance during OPC [22,23]. Therefore we monitored the expression of IL-17A in Foxp3+ T_regs_ in the same OPC infection model by orally infecting the Foxp3^GFP^ reporter mice with *C. albicans*. On day 2 after infection, we isolated the cells from spleen, lymphnodes and mouse oral lamina propria and intraepithelial cells (MOIL) isolated from tongue and palatal tissues [26]. Both CD4^+^Foxp3^−^ effector cells and CD4^+^Foxp3^+^ T_regs_ responded to infection by expressing IL-17A in the draining axillary lymph nodes (ALN), cervical lymph nodes (CLN) and MOIL in infected mice (Figure 1a). Although there were a few Foxp3+IL-17A+ cells in sham infected mice, CD44^high^ (activated) CD4 cells showed a substantial increase in Foxp3+IL-17A+ cells only in infected mice. However, there was no increase of these cells in spleen (Figure 1a). We further confirmed the induction of Foxp3+IL-17A+ cells by gating on CD4^+^Foxp3^+^ cells only (Figure 1b,c). Although a fraction of T_regs_ produced IL-17A on d1 and d2 after infection as shown previously [22], T_regs_ still increased IL-17A in effector cells, decreased fungal burden, and modulated immunopathology and weight loss in T_reg_ recipients during OPC on d5 (data not shown, [22]).

Human mucosal Foxp3+T_regs_ express IL-17A in inflammatory conditions, such as periodontitis, psoriasis and IBD [4]. Therefore, we next tested whether IL-17A^+^Foxp3^+^ cells are induced during IBD in mice. We adoptively transferred CD45.1 Th17 cells into CD45.1 Rag1^−/−^ mice to initiate IBD [22,27]. Two weeks after IBD induction, we injected CD4^+^CD25^+^GFP^+^ T_regs_, derived from congenic CD45.2 Foxp3^GFP^ reporter mice. Five days after T_reg_ injection, we isolated spleen, mesenteric lymph nodes (MLN) and mouse gut intraepithelial and lamina propria leukocytes (MGIL) and measured the IL-17A induction upon restimulation *in vitro*. We gated on CD4+Foxp3^−^CD45.1+effector cells and CD4+Foxp3^+^CD45.1-T_regs_ to examine IL-17A induction in these cells. We observed that T_regs_ were able to produce IL-17A, especially in MLN and LP during inflammation *in vivo* (Figure S1a). Despite IL-17A production in T_regs_, the injected T_regs_ still modulated IBD and weight loss in T_reg_ recipients (Figure S1b). Taken together, these data demonstrate that infections and inflammatory conditions can induce IL-17A production in a fraction of Foxp3+T_regs_ *in vivo*, but do not affect their immunomodulatory functions.

### 2.2. TLR-2 Ligands Directly Induce Proliferation of T_regs_ Independently of TCR Activation

**Figure 2:**
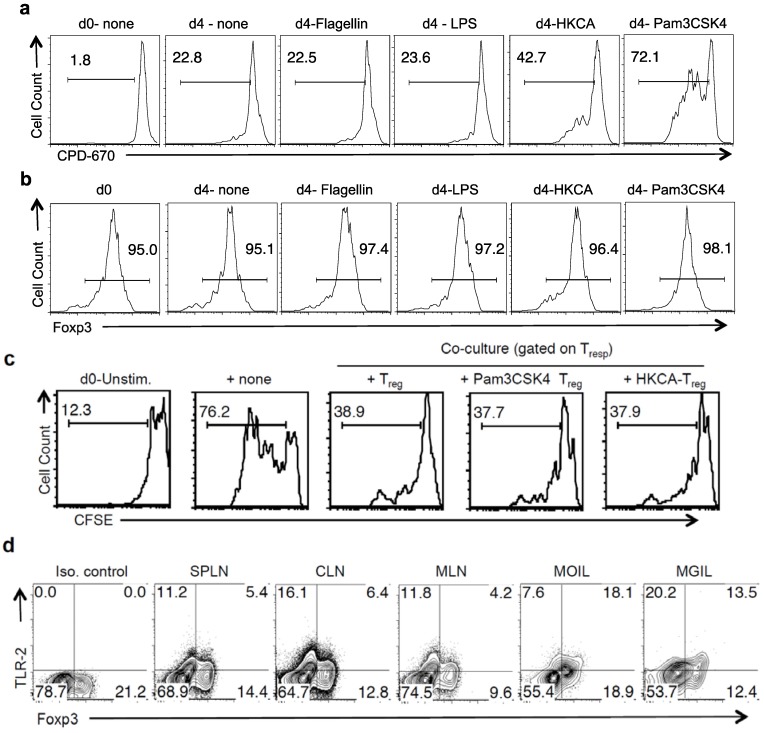
T_regs_ proliferate in response to TLR ligands and IL-2 independently of TCR. (**a**) CPD-670 dilution (proliferation) of T_reg_ cells, with indicated TLR ligands added at the beginning of stimulation; (**b**) Histograms of Foxp3 expression of live T_regs_ that were stimulated as in “a”; (**c**) T_regs_ stimulated with or without TLR-2 ligands as in “a”, were isolated and washed after two days. They were restimulated with fresh CFSE-labeled CD4 naive responder (T_resp_) cells in co-cultures. Some T_resp_ cells were not stimulated (d0-unstim) or stimulated alone (+ none). T_resp_ proliferation is shown; (**d**) Foxp3 and TLR-2 expression in CD4 T cells isolated *ex vivo* from indicated tissues. (**a**–**d**) represent three independent experiments.

As *C. albicans* activates TLR-2 and dectin signaling that promote Th17 responses [28], we examined whether TLR-2 ligands and dectin ligands induced IL-17A in T_regs_ *in vitro*. We sought to investigate the effects of TLR-2 ligands on highly purified CD4^+^CD25^+^Foxp3^+^ T_regs_ under non-inflammatory and inflammatory conditions. First we examined whether TLR ligands impacted T_regs_ in the absence of TCR activation, but with IL-2 alone. IL-2 stimulation of T_regs_ in the absence of TCR ligation would be akin to non-inflammatory conditions and would induce homeostatic proliferation in T_regs_ [29,30,31]. We isolated CD4^+^ CD25^+^Foxp3^GFP+^ cells (T_reg_) and control CD4^+^CD25^−^Foxp3^GFP−^CD44^l^°^w^ naïve T cells (T_con_) from Foxp3^GFP^ reporter mice (Figure S2), and stimulated them with IL-2 [30]. This condition also prevented the proliferation of possible contaminating T_cons_ among T_regs_, which expand vigorously with TCR stimulation in cultures. We labeled T_regs_ with cell proliferation dye-670 (CPD-670) and tested their viability and proliferation with and without various TLR ligands. As expected, naïve T_con_ cells died under these conditions in the absence of TCR activation (data not shown). However, IL-2 stimulation alone maintained T_reg_ viability by virtue of constitutive IL-2 receptor expression on T_regs_. On day 4 after stimulation, heat killed *Candida albicans* (HKCA), a natural Tlr-2/Dectin ligand, and Pam3CSK4, a TLR2/6 ligand increased the proliferation of T_regs_ (Figure 2a). Other TLR ligands, such as Poly A:U (TLR-3) (data not shown), LPS(TLR-4) and Flagellin (TLR-5), showed no effect (Figure 2a). As TLR-2 ligands have been shown to transiently reduce Foxp3 mRNA expression of TCR activated T_regs_ [15], we measured Foxp3 expression of T_regs_. HKCA and Pam3CSK4 did not reduce Foxp3 expression in T_regs_ (Figure 2b). To examine their suppressive capacity, we washed the T_regs_ to remove the TLR-2 ligands, and stimulated them with α-CD3 and APC along with freshly isolated, carboxyfluorescein di-acetate succinimidyl ester (CFSE) labeled CD4^+^CD25^−^ responding cells (T_resp_) in co-cultures. T_resps_ that were stimulated alone without T_regs_ showed increased proliferation (76.2%) compared to T_resps_ that were stimulated with T_regs_ (38.9%) on day 4 after stimulation (Figure 2c). T_regs_ that were previously stimulated with IL-2 alone, or with Pam3CSK4 or HKCA, were capable of suppressing the proliferation of T_resps_ (Figure 2c). These data show that TLR-2 ligands, along with IL-2 can induce proliferation of T_regs_ in the absence of TCR signals, without affecting their suppressive capacities. Because T_regs_ responded to TLR-2 ligands without TCR activation, we hypothesized that they might express TLR-2 protein *ex vivo*. We found that both Foxp3^−^ and Foxp3^+^ CD4+ cells from spleen and lymph nodes expressed TLR-2 *ex vivo* (Figure 2d)*.* Interestingly, TLR-2 expression was markedly increased in mucosal T_regs_ found among MOIL and mouse gut intraepithelial and lamina propria cells (MGIL), implying an important role of TLR-2 in mucosal T_regs_.

### 2.3. TLR-2 Ligand Mediated Proliferation in T_regs_ is Directly Dependent on TLR-2 Expression on T_regs_

**Figure 3:**
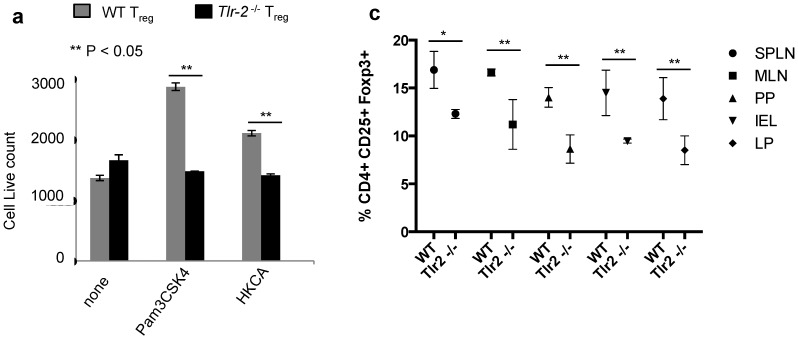

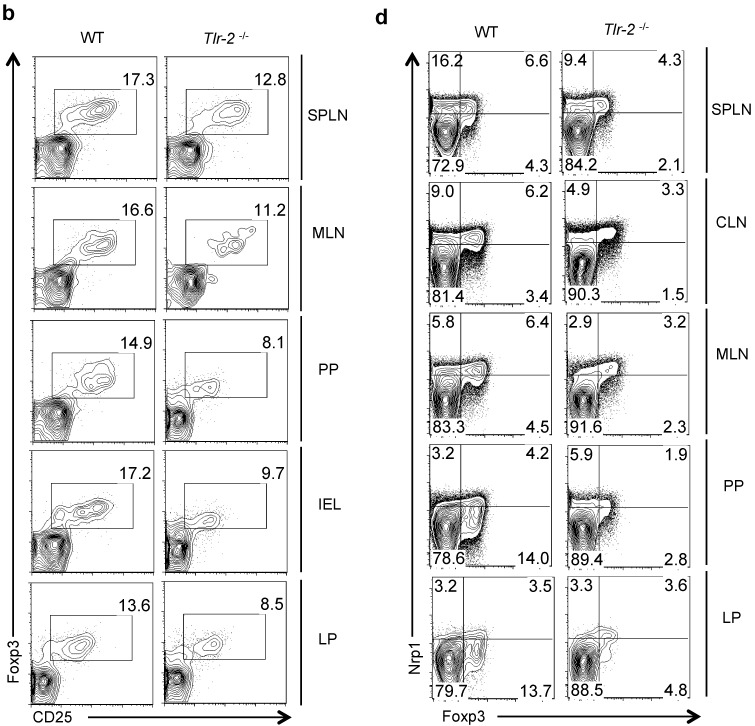
Pam3CSK4 and HKCA induce proliferation of T_regs_ in a TLR-2 dependent manner. (**a**) Live cell counts (PI^neg^ and FSC^high^) of T_reg_ cells from WT (grey) or Tlr-2^−/−^ (black) mice stimulated with various TLR-2 ligands for three days as in “2a”. CD4^+^CD25^+^Foxp3^+^ (**b**), or CD4^+^ Foxp3^+^Nrp1^+^ (**d**), frequencies in WT (left) and Tlr-2^−/−^ (right) cells isolated *ex vivo*. (**c**) Statistical analyses showing T_reg_ frequencies in WT and Tlr-2^−/−^ mice, as determined in “b”.

We then sought to confirm that proliferation induced by TLR-2 ligands was dependent on TLR-2 expression in T_regs_. We isolated T_regs_ from the spleens of WT or Tlr-2^−/−^ mice and stimulated with IL-2 and TLR-2 ligands. On day 3 after stimulation, HKCA and Pam3CSK4 increased the T_reg_ cell numbers in WT T_regs_ but not in Tlr-2^−/−^ T_regs_, showing that they induced proliferation in TLR-2 dependent manner in T_regs_ (Figure 3a). If TLR-2 signaling induced proliferation of T_regs_ in the absence of TCR ligation or inflammatory cytokines, TLR-2 agonists in gut commensal microbes and other endogenous ligands may also promote T_reg_ proliferation under homeostatic conditions *in vivo*. To test this idea, we examined the frequency of CD4+CD25+Foxp3+ T_regs_ in various organs, including SPLN, MLN, payer’s patches (PP), gut lamina propria (LP), and the gut intra epithelial lymphocytes (IEL) in WT and Tlr-2^−/−^ mice, using flow cytometry. We found that the frequency of CD4+CD25+Foxp3+ cells was significantly reduced in Tlr-2^−/−^ mice when compared to the WT mice (Figure 3b,c). A previous study showed that the frequency of CD4^+^CD25^+^ cells was lower in the peripheral blood of Tlr-2^−/−^ mice than in WT mice [21]. However, neither Foxp3 expression nor the frequency of thymically derived T_regs_ (tT_regs_) and peripheral T_regs_ (pT_regs_) was examined. Therefore we evaluated the expression of neuropilin-1 (Nrp-1), a marker on tT_regs_ in peripheral organs and mucosal tissues. The frequency of both the T_reg_ populations were decreased slightly in SPLN, but dramatically in CLN, MLN, PP and LP of Tlr-2^−/−^ mice, when compared to the WT mice (Figure 3d). Interestingly, in payer’s patches and the intestinal lamina propria, the reduction of T_regs_ in Tlr-2^−/−^ mice was more pronounced in the Nrp-1^−^ pT_reg_ compartment (Figure 3d). These results highlight the importance of TLR-2 ligands in promoting tT_reg_ and pT_reg_ homeostatic proliferation under steady-state conditions *in vivo*.

### 2.4. TLR-2 Ligands Did Not Reduce Foxp3 Expression, but Induced IL-17A in Foxp3+ T_regs_ under Th17 Conditions *in Vitro*

**Figure 4:**
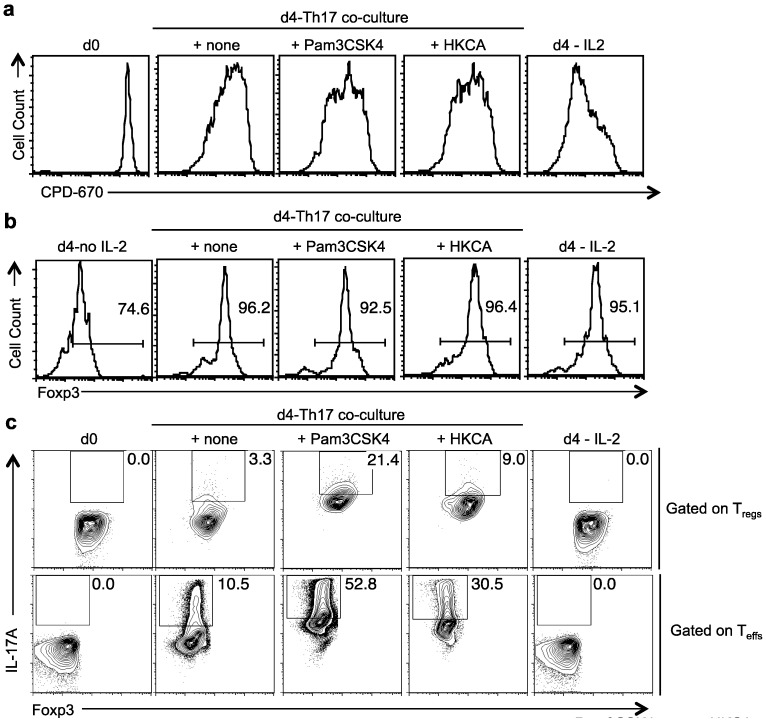

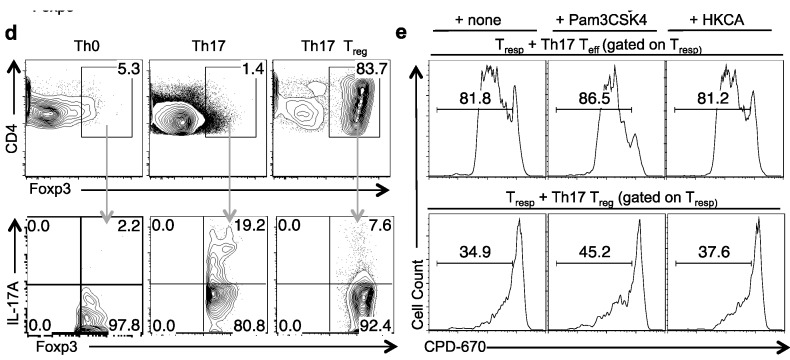
TLR-2 ligands induce proliferation but not plasticity in T_regs_ stimulated under Th17 conditions. CPD-670 dilution (proliferation) (**a**), or Foxp3 expression (**b**), of T_reg_ cells co-cultured with conventional CD4+ T cells under Th17 conditions for four days, with or without Pam3CSK4, HKCA, or by themselves without (d4-no IL-2) or with IL-2 (d4-IL-2). CD45.2 T_regs_ are gated in the analyses. (**c**) T_regs_ were stimulated as in “a”. Foxp3 and IL-17A expression of cells gated on CPD670+CD45.2 T_regs_ (upper panel), or CPD670-Foxp3-CD45.1 T_eff_ (lower panel) in co-cultures. (**d**) Foxp3 and CD4 expression (upper panel), of naïve cells that were stimulated under Th0 (Th0) or Th17 (Th17) conditions, or T_regs_ that were stimulated under Th17 (Th17 T_reg_) conditions. Foxp3 and IL-17A expression (lower panel), of the Foxp3+ cells in the upper panel. (**e**) Proliferation suppression of the CD4 responder cells (as in “2c”) co-cultured with GFP^+^ T_reg_ cells isolated from cultures stimulated as in “a”. Data represent three independent experiments.

We then sought to investigate whether TLR-2 ligands may promote IL-17A in T_regs_ stimulated under Th17 inducing conditions *in vitro.* Although TLR-2 ligands have been shown to reduce Foxp3 expression and suppressive properties in T_regs_ [15], whether IL-17A is induced in T_regs_ is unknown. However, Strober and colleagues have shown that T_regs_ produce IL-17A in Th17 inducing conditions *in vitro* [32]. Therefore, we stimulated T_regs_ in co-cultures along with T_cons_ at a ratio of 1:10 under Th17 inducing conditions. T_con_ cells proliferated in to Th17 effectors (T_eff_). As controls, we stimulated T_regs_ in Th0 conditions. To distinguish T_regs_ and T_eff_ in co-cultures, we labeled T_regs_ using CPD670 in addition to CD45.2 congenic marker on T_regs_ in co-cultures (Figure S3a). We gated on CPD670^+^CD45.2^+^T_regs_ (Figure S3a), and examined their proliferation, Foxp3 expression and IL-17A expression. Four days after stimulation, Pam3CSK4 and HKCA increased the proliferation of T_regs_ (Figure 4a). Although ~20% of the cells lost Foxp3 expression completely, many of these cells were non-proliferating T_regs_, which did not dilute CPD670 (Figure S3b). These cells were found in the presence and the absence of TLR-2 ligands (Figure S3b), likely due to the fact that IL-6 that can transiently reprogram T_regs_ to lose Foxp3 expression [22,33]. However, when proliferating T_regs_ were examined, TLR-2 ligands did not lead to reduction in Foxp3 expression (Figure 4b). As expected, in the absence of IL-2, T_regs_ showed poor survivability and reduced Foxp3 expression ([30], Figure 4b). We also observed that a small fraction of the Foxp3+ cells (~3%) consistently expressed IL-17A (Figure 4c). However, TLR-2 ligands increased the frequency and the levels of IL-17A substantially (Figure 4c, upper panel). We did not observe IL-17A expression in control Th0 stimulated T_regs_ (Figure 4c, upper panel). To verify the specificity of the staining, we used T_regs_ from *Il17a^-/-cre^* mice, which barely showed IL-17A expression (Figure S4). Under Th0 and Th17 polarizing conditions, a small fraction of T_effs_ also induced Foxp3 expression (iT_regs_) (Figure 4d, upper panel). iT_regs_ that were induced in Th0 conditions did not express IL-17A, while iT_regs_ induced under Th17 polarizing conditions up-regulated IL-17A, similar to natural T_regs_ (Figure 4d). These data show that both *in vitro* induced iT_regs_ and natural T_regs_ isolated from mice are capable of producing IL-17A under Th17 inducing conditions.

As we have shown previously, T_regs_ did not suppress effector Th17 cells (T_eff_) because of IL-6 and excess IL-2 in Th17 polarizing milieu [22], both in the presence or absence of TLR-2 ligands (data not shown). We then sought to determine whether T_regs_ retain their suppressive activity after the withdrawal of Th17 cytokines and TLR-2 agonists. Therefore we stimulated T_regs_ under Th17 inducing conditions, in the presence or absence of Pam3CSK4 and HKCA. After 4 days, we washed the T_regs_ (Th17 T_regs_) and co-cultured them with CPD-670 labeled CD4^+^CD25^−^naïve responder cells (T_resp_), in the presence of α-CD3 and APC [34]. As controls, we used T_effs_ (Th17 T_eff_) that were previously stimulated under Th17 inducing conditions in the presence or absence of TLR-2 ligands, and co-cultured them with T_resps_. As determined by T_resp_ CPD-670 dilution, we found that Th17 T_regs_ stimulated with TLR-2 agonists were capable inducing proliferation suppression in T_resps_ (Figure 4e). These results demonstrate that T_regs_ produced IL-17A and lost their suppressive capacity only transiently in the presence of Th17 cytokines in the milieu. However, they were capable of suppressing CD4+ T cells upon pro-inflammatory cytokine and TLR-2 ligand withdrawal.

**Figure 5:**
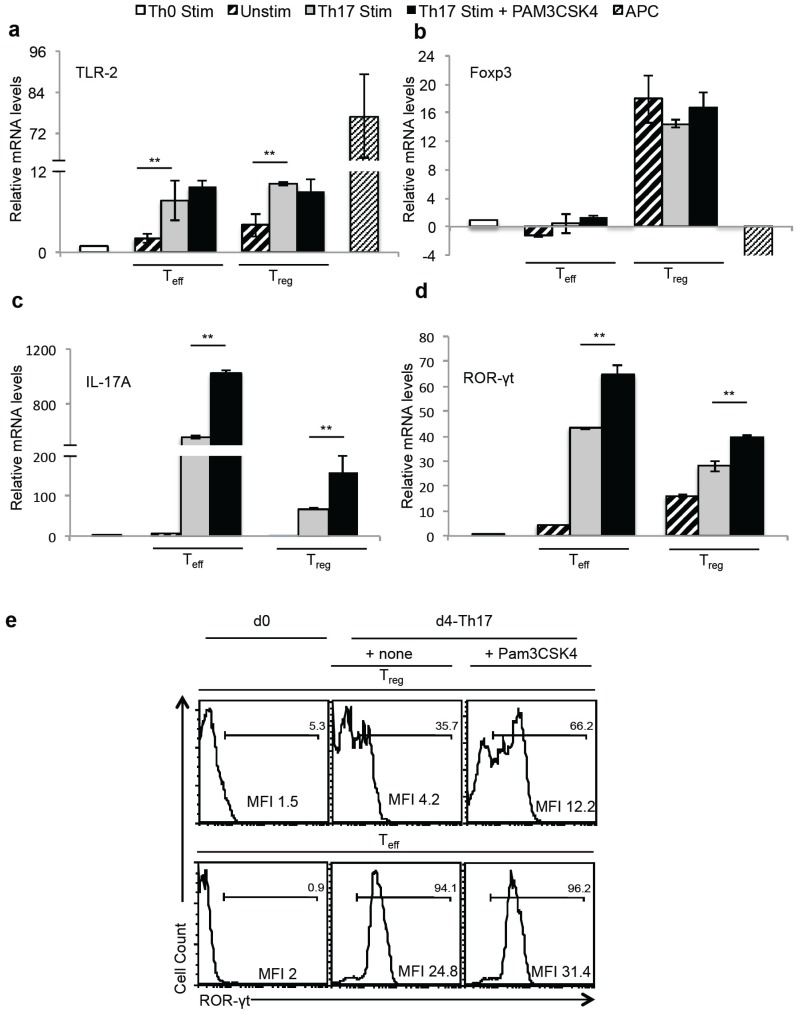
T_regs_ up-regulate TLR-2, Foxp3, IL-17A and ROR-γt in the presence of Pam3CSK4 and Th17 conditions *in vitro*. Naïve cells or T_regs_ were stimulated under Th0 (Th0 stim) or Th17 (Th17 stim) conditions as in “4d”, with or without Pam3CSK4. Unstimulated CD4 T cells (Unstim) and CD90 depleted APC activated with Pam3CSK4 were used as controls (APC). Two days later, RNA was isolated from CD90 sorted T cells from Th17 cultures, and relative mRNA levels of Tlr-2 (**a**), Foxp3 (**b**), IL-17A (**c**) and ROR-γt (**d**) were assessed using q-PCR. (**e**) ROR-γt expression of the cells, gated on Foxp3+ CD45.2 T_regs_ (upper panel), or Foxp3-CD45.1 T_eff_ (lower panel) in the co-cultures is shown. Geometric mean fluorescence intensities (MFI) are shown.

To further validate the role of TLR-2 ligands in induction IL-17A in Foxp3^+^T_regs_, we assessed the relative mRNA levels of *Tlr-2*, *Foxp3*, *IL-17A* and *ROR-γt* on d2 after stimulation. We used Th0 stimulated CD4^+^CD25^−^ Foxp3^GFP−^ cells as the normalizing control, unstimulated CD4^+^CD25^−^ Foxp3^GFP−^ cells, and CD90 depleted APC as additional controls. Compared to Th0 cells, both CD4 effector cells and T_regs_ had higher levels of *Tlr-2* mRNA expression under Th17 inducing conditions (Figure 5a). As expected, Foxp3 mRNA expression was observed only in T_regs_ and was unchanged between un-stimulated T_regs_ and Th17 T_regs_ that were stimulated with and without Pam3CSK4 (Figure 5b). However, Th17 T_regs_ expressed IL-17A and ROR-γt that were further up-regulated by Pam3CSK4 (Figure 5c,d). Similar to mRNA expression, T_regs_ stimulated with Pam3CSK4 showed ROR-γt protein up-regulation, although at lower levels (MFI ~4–12) when compared to T_eff_ (MFI ~24–31) (Figure 5e). Taken together, these results validate that although TLR-2 ligands induced proliferation and increased IL-17A and ROR-γt expression among Foxp3^+^T_regs_, they do not reprogram the cells or affect the stability of Foxp3 expression.

### 2.5. TLR-2 Ligands Induced IL-17A Production Is Only Partially Dependent on TLR-2 Expression in T_regs_

**Figure 6:**
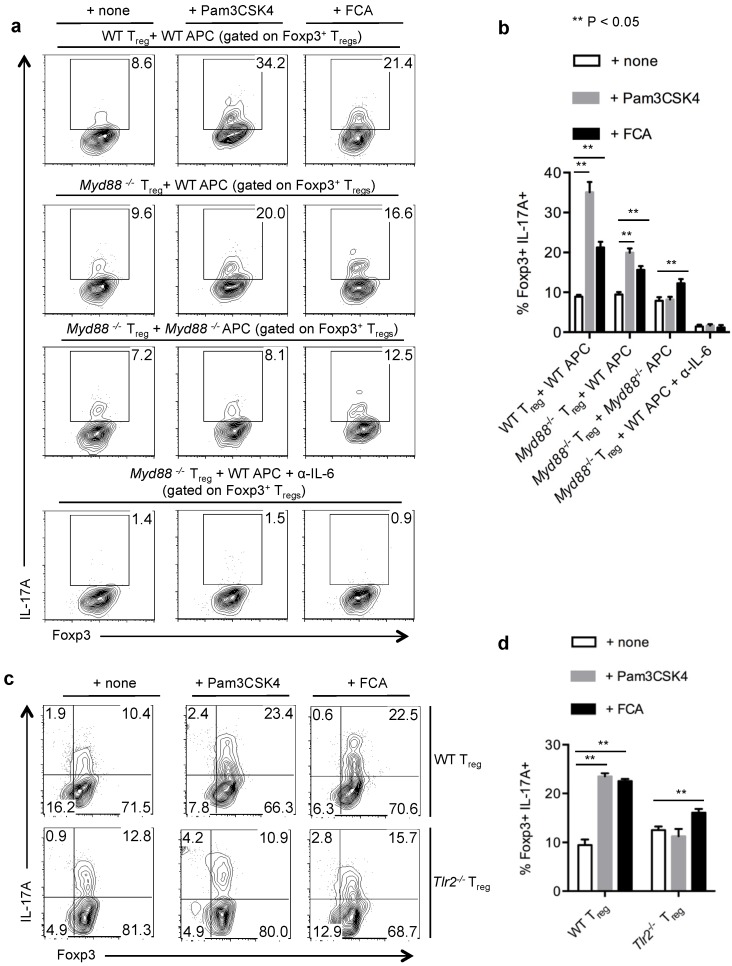
IL-17A production of T_regs_ in an inflammatory milieu is dependent on TLR-2 expression in T_regs_ and APC. (**a**,**b**) WT or Myd88^−/−^ T_regs_ were stimulated with WT APC or Myd88^−/−^ APC as indicated, without or with Pam3CSK4 or fixed *Candida albicans* (FCA) germ tube for four days. Foxp3 and IL-17A expression (**a**), and statistical representation of IL-17A expression in Foxp3+ T_regs_ (**b**). α-IL-6 antibodies were added under Th17 conditions without exogenous IL-6 (**a**, bottom panel). (**c**,**d**) WT or Tlr-2^−/−^ T_regs_ were stimulated with Tlr-2^−/−^ APC, without or with Pam3CSK4 or FCA for 4 days. Foxp3 and IL-17A expression in CD4 gated cells (**c**), and statistical representation of IL-17A expression (**d**), are shown. Data represent at least 3 independent experiments.

We next investigated the requirement of TLR-2 receptor in induction of IL-17A in Foxp3^+^T_regs_. Myd88 is the critical adaptor molecule downstream of TLR-2 ligand stimulation [35], and therefore we examined whether Myd88 is required for IL-17A up-regulation. We isolated WT or *Myd88*^−/−^ T_regs_ and stimulated them under Th17 inducing conditions in the presence of WT APC with or without Pam3CSK4 and formaldehyde fixed *Candida albicans* hyphae (FCA). We found that the absence of Myd88 signaling in T_regs_ slightly reduced TLR-2 dependent IL-17A production in the presence of WT APC (Figure 6a, top two panels, 6b). We then stimulated *Myd88*^−/−^ T_regs_ with *Myd88*^−/−^ APC in the presence or absence of TLR-2 ligands. Absence of Myd88 in APC further reduced the IL-17A induction almost to the basal levels in T_regs_ (Figure 6a, third panel, 6b). Previous studies have shown TLR activation in APC can promote IL-6, a crucial cytokine for IL-17A induction in CD4 T cells [16,19]. Accordingly, when we stimulated *Myd88*^−/−^ T_regs_ with WT APC in the presence of α-IL-6 neutralizing antibody, IL-17A was completely abolished in T_regs_ (Figure 6a, bottom panel, 6b). To further validate the direct TLR-2 signaling requirement in induction of IL-17A in T_regs_, independent of TLR-2 expression in APC, we stimulated WT and *Tlr-2*^−/−^ T_regs_ with *Tlr-2*^−/−^ APC under Th17 inducing conditions. Only a fraction of both WT and *Tlr-2*^−/−^ T_regs_ produced IL-17A (Figure 6c,d). However, Pam3CSK4 further promoted IL-17A expression only in WT T_regs_ but not in *Tlr-2*^−/−^ T_regs_. In the presence of FCA, IL-17A induction was only partially abrogated in *Tlr-2*^−/−^ T_regs_, showing that FCA mediated effects were only partially dependent on TLR-2 expression (Figure 6c,d). Taken together, these data show that under Th17 inducing conditions, maximal induction of IL-17A requires both direct and indirect TLR-2/Myd88 signaling on T_regs_, as well as APC. IL-6 was a critical factor in the milieu for T_regs_ to produce IL-17A both in the presence and the absence of TLR-2 ligands.

### 2.6. Direct TLR-2 Signaling in T_regs_ is Required for Maximal Induction of Foxp3^+^ IL-17A+ Cells during OPC *in Vivo*

**Figure 7:**
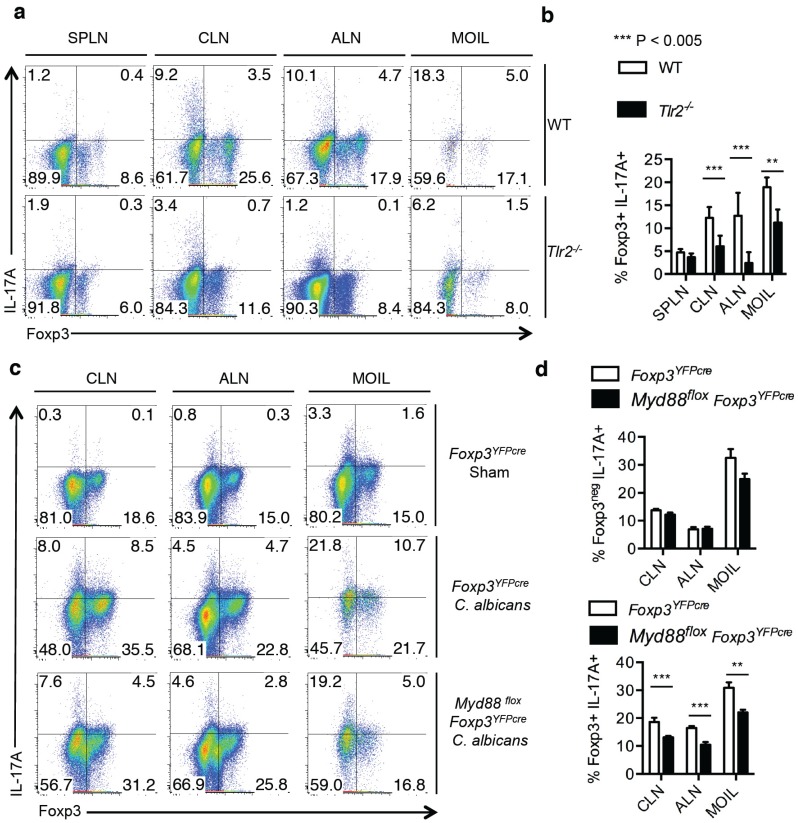
IL-17A expression in CD4^+^ Foxp3^+^ T_regs_ requires TLR-2 signaling in T_regs_ during *C. albicans* infection *in vivo*. WT C57BL/6 or Tlr-2^−/−^ mice infected with *C. albicans*, and tissues were isolated as in “1a”. Flow cytometric plots gated on CD4+ cells showing IL-17A expression (**a**), and statistical analyses of IL-17A expression in CD4+Foxp3+ cells (**b**) are shown. Data represent triplicate experiments. (**c**,**d**) Foxp3^YFPcre^ or Myd88 ^fl^°^x^ Foxp3^YFPcre^ mice were infected, and cells were isolated as in “1a”. Flow cytometric plots gated on or CD4+ cells showing IL-17A expression (**c**), and statistical analyses of IL-17A expression in CD4+Foxp3-cells (**d**, upper panel) and CD4+Foxp3+ cells (d, lower panel) are shown.

Lastly, we examined the requirement of TLR-2 expression for inducing IL-17A in T_regs_ during OPC *in vivo*. Although TLR-2 signaling promotes T_reg_ proliferation during systemic candidiasis, IL-17A induction was not assessed in T_regs_ during the infection [21]. To determine the role of TLR-2 signaling in induction of IL-17A in Foxp3+T_regs_* in vivo*, we orally infected WT and *Tlr-2^−/−^* mice and measured IL-17A production in CD4+ T cells. We found reduced frequency of T_regs_ in *Tlr-2^−/−^* mice compared to WT mice infiltrating the draining lymphnodes and MOIL during infection (Figure 7a). Importantly, among those Foxp3+CD4+ T_regs_ the frequency of IL-17A producers was at least three to five times decreased in *Tlr-2^−/−^* mice than in WT mice (Figure 7a,b). These data show that TLR-2 signaling enhances proliferation and maximal IL-17A induction in T_regs_. Consistent to the role of TLR-2 signaling in promoting IL-17A in effector CD4 cells [14], we also found reduced IL-17A production among Foxp3-effector CD4+ cells in *Tlr-2^−/−^* mice, when compared to the WT mice (Figure 7a). To further confirm the direct role of TLR signaling in T_regs_, we used *Myd88^fl^°^x^ Foxp3^YFPcre^* mice, in which *Myd88* gene was conditionally deleted in Foxp3^+^ cells. Compared to the control *Foxp3^YFPcre^* infected mice, the induction of IL-17A was significantly reduced in *Myd88^fl^°^x^ Foxp3^YFPcre^* mice in Foxp3^+^ cells (Figure 7c,d lower panel). Although there was a slight decrease in IL-17A production even in the effector cells in MOIL, the frequency of IL-17A producing effector cells was unchanged between the groups in CLN and ALN (Figure 7c,d upper panel). Taken together, these results reveal that direct TLR-2 signaling in T_regs_ plays a crucial role in increasing IL-17A+Foxp3+ T_regs_ during OPC *in vivo*.

## 3. Discussion

Foxp3+T_regs_ produce little or no IL-17A in a sterile environment such as thymus in humans or in naïve mice raised under specific pathogen free conditions [6]. Voo *et al.*, were one of the first groups to show the enrichment of peripherally generated IL-17A+T_regs_ in tonsils, compared to blood, in individuals undergoing tonsillectomy [6]. As tonsils constitute an oral mucosal environment, we hypothesized that oral infections may lead to the generation of these IL-17A+Foxp3+ cells. To verify the tenet, we examined whether they could be induced during oropharyngeal candidiasis infection in mice. Our study shows that OPC can induce Foxp3+IL-17A+T_regs_ (T_reg_-17) in a TLR-2/Myd88 dependent manner, revealing one of the mechanisms by which T_reg_-17 cells are induced during inflammation. We also show that while TLR-2 ligands promote only proliferation in the context of non-inflammatory homeostatic conditions, they can increase proliferation and IL-17A production in T_regs_ in the context of APC and inflammatory conditions. Although some of these data are in line with the reports that showed TLR-2 signaling induces survival and proliferation in T_regs_ activated by TCR [12,17], our study also unveils a novel function of TLR-2 in IL-2 dependent homeostatic proliferation of T_regs_. Reduced tT_regs_ and pT_regs_ in *Tlr-2*^−/−^ mice demonstrate that TLR-2 ligands from commensals play a role in maintaining their steady state proliferation (Figure 3b,d). Although TLR-2 signaling was not examined, defective T_regs_ in germfree and antibiotic treated mice in previous studies [36,37,38,39,40] further confirm the role of commensals in maintaining T_regs_ in the gut. Further strengthening the idea that TLR-2 signaling induces proliferation of T_regs_ *in vivo*, and similar to a previous study [21], we observed reduced T_regs_ at the site of infection in *C. albicans* infected *Tlr-2*^−/−^ mice compared to WT mice. CD4 effector cells and T_regs_ showed higher TLR-2 protein expression in Th17 inflammatory conditions, compared to Th0 cultures (Figure 5a), showing that Th17 inflammatory cytokines may promote enhanced TLR-2 signaling and proliferation in CD4+T cells [14]. However, contrary to previous findings [15], TLR-2 ligands did not lead to loss of Foxp3 expression. Although under Th17 inflammatory milieu, T_regs_ did not suppress CD4+ cells, and lost Foxp3 transiently [22], T_regs_ that expanded with TLR-2 agonists were capable of suppressing fresh CD4+ T cells, upon IL-6 withdrawal. These data imply that the reversal of suppression by TLR-2 agonists in previous studies is likely due to the cytokines in effector CD4 T cells and APC induced by TLR-2 ligands, but not due to their direct effect on T_regs_ [15]. Apparent loss of Foxp3 expression could also be due to the usage of CD4^+^CD25^+^ cells in some studies, instead of CD4^+^CD25^+^Foxp3^+^ cells, that might have led to proliferation of Foxp3^−^ cells in their cultures.

Although TLR-2 ligands can directly induce proliferation of T_reg_-17 cells, maximal IL-17A induction in T_regs_ requires IL-6, and TLR-2/Myd88 signaling in APC as well. Mechanistically, although it is conceivable that IL-6 may prime IL-17A induction in Foxp3+T_regs_ [6], we report here for the first time the role of TLR-2 signaling in increasing the frequency of T_reg_-17 cells, by promoting proliferation of IL-17A producing Foxp3+T_regs_ *in vitro* and *in vivo*. However, the lower levels of ROR-γt and IL-17A that we observed in T_regs_ when compared to the effector cells, is likely due to the inhibitory effect of Foxp3 on ROR-γt expression [41,42]. In the context of *Candida albicans*, even in the absence of TLR-2 signaling in both T_regs_ and APC, FCA could partially induce IL-17A in T_regs_. We speculate that dectin-1 or 2 signaling is also involved in the induction of IL-17A by *C. albicans* [43]. However, our results showing diminished production of IL-17A in effector CD4+ cells and T_regs_ in *Tlr-2*^−/−^ mice*,* underscore the importance of *Candida* derived TLR-2 ligands, in orchestrating effector functions and induction of T_reg_-17 cells during OPC. T_reg_-17 cells that we describe herein can be induced both from a fraction of tT_regs_ and iT_reg_ cells induced in cultures (Figure 4d). Similarly, T_reg_-17 cells are possibly derived from tT_regs_ and pT_regs_ during OPC *in vivo* as well. Whether these T_reg_-17 cells also play roles in direct antifungal Th17 responses remain to be investigated. This tenet would be consistent with the requirement of T_regs_ in protective immunity to fungi [24]. However, in the context of suppressing inflammation, Foxp3+ T_regs_ undergo effector specific functional adaptation in order to suppress distinct effector responses[44]. For example, T_regs_ acquire effector Tfh characteristics such as Bcl6 and CXCR5 expression to regulate Tfh and germinal center responses in a Bcl-6 dependent manner [45,46]. Likewise, they require IRF-4 and STAT-3 signaling to suppress Th2 and Th17 immunopathology, respectively, *in vivo* [44,47]. However, it is unknown if T_regs_ with active STAT-3 signaling express IL-17A to adapt to their environment and engage distinct effector response—specific suppression of Th17 immunopathology. If this is the case, T_reg_-17 cells promoted by TLR-2 ligands and Th17 cytokines may be a specific population of T_regs_ programed for antimicrobial functions at early time-points, and for controlling Th17 pathology at later phases of infection. Supporting this notion, the T_reg_ population, which included the effector T_reg_-17 cells, while enhancing IL-17A in Th17 cells initially, was capable of suppressing OPC immunopathology and IBD immunopathology at later time points in our studies ([22], Figure S1b). Also *in vitro*, despite acquiring effector functions, >90% of CD25+Foxp3+ T_regs_ had stable Foxp3 expression, and retained their suppressive properties, upon IL-6 and TLR-2 ligand withdrawal (Figure 4e). Taken together, these data provide important insights into the role of TLR-2 signaling in promoting T_reg_-17 populations without affecting their suppressive properties during inflammation and infections. We believe that during mucosal inflammation, TLR-2 ligands from commensals and pathogens may drive effector cytokine production in T_regs_. Although in the context of local IL-6, T_regs_ lose their suppressive functions and produce effector cytokines transiently, TLR-2 ligands do not lead to their terminal differentiation in to effector cells. Future studies are warranted to further examine the precise functions of T_reg_-17 cells during infections and homeostasis in mucosal environments in human diseases.

## 4. Materials and Methods

### 4.1. Mice

C57BL/6, Balb/C, C57BL/6 WT, *Tlr-2*^−/−^, *Il17a*^−/−*cre*^ and *Myd88*^−/−^, *Foxp3^GFP^* reporter mice, *Foxp3^YFPcre^* reporter mice and *Myd88^fl^°^x^* mice were purchased from Jackson Laboratories and were used at 8–12 weeks of age. *Myd88^fl^°^x^ Foxp3^YFPcre^* mice were generated by breeding *Foxp3^YFPcre^* reporter mice and *Myd88^fl^°^x^* mice, and *Foxp3^YFPcre^* littermates were used as controls. All the mice were maintained in the Animal Resource Center at Case Western Reserve University and cared for in accordance with institutional guidelines and approved IACUC protocols.

### 4.2. Reagents and Antibodies

Purified α-CD3 (145-2C11), purified α-CD28, α-CD25 (3C7), Biotin α-CD25, fluorescein isothiocyanate (FITC)-α-CD4, phycoerythrin (PE)-α-CD25, unconjugated, (allophycocyanin) APC, Alexafluor-647 or PE conjugated α-CD44, APC, Alexafluor 647, PE or PE-Cy7 conjugated IL-17-A, Foxp3 antibodies, IL-6 blocking antibodies, α-IL-4 and IFN-γ antibodies, were all purchased from eBiosciences (San Diego, CA). Mouse CD4 isolation kit II was purchased from Miltenyi Biotec (Auburn, CA). EasySep isolation kits were purchased from Stem Cell technologies (Canada). Human IL-2 purchased from NCI, was a kind gift from Mike Lenardo lab (NIAID, NIH). Recombinant mouse IL-6 was purchased from BioBasic Inc (Amherst, NY), and human TGF-β was purchased from R&D Systems (Minneapolis, MN). Mouse cells were cultured in complete RPMI-1640 (Bio-Whittaker) supplemented with 10% FCS, 100 U/mL penicillin, 100 μg/mL streptomycin, 5 mM Glutamax (Invitrogen) and 50 μM β-mercaptoethanol.

### 4.3. Cell Purification

Splenocytes and lymphnodes were harvested from 8- to 12-week-old mice and pooled. CD4 cells were MACS (magnetic assisted cell sorting) purified by using CD4 isolation kit II (Miltenyi Biotec, Auburn, CA) or sorted using EasySep kits. Purified CD4 cells were FACS (Fluorescence Activated Cell Sorting) sorted for CD4^+^CD25^−^GFP^−^ T_con_ cells or CD4^+^CD25^+^ GFP^+^ T_reg_ cells (>99% purity). In some experiments CD25^+^ T_regs_ and CD4^+^CD25^−^ CD44^l^°^w^ naïve T_con_ cells were sorted using EasySep kits, and the purity of T_regs_ was more than 92%. 6 × 10^4^ FACS sorted T_cons_ or T_regs_ were cultured in U-bottom 96 well plates with 2 ng/mL IL-2 only, or also with TLR ligands. Pam3CSK4, heat killed *Candida albicans* (HKCA), Lipopolysaccharide (LPS), and Flagellin were purchased at Invivogen and were used at following concentrations: LPS, 1 ug/mL; Flagellin, 1μg/mL; Pam3CSK4, 5 μg/mL and HKCA, 10^6^/mL. In some experiments, formaldehyde fixed *Candida albicans* germtube (FCA) were used at 10^6^/mL.

### 4.4. Th17 Differentiation

T_reg_ cells were cultured alone in U-bottom 96 well plates, or co-cultured with T_cons_ in the presence of soluble 1 μg/mL of α-CD3 and 2 μg/mL α-CD28 antibodies under Th17 inducing conditions for 3–4 days. Th17 cells were polarized using recombinant mouse IL-6 (25 ng/mL), recombinant human TGF-β (2 ng/mL), recombinant mouse IL-23 (20 ng/mL), recombinant mouse IL-1β (20 ng/mL), α-IFN-γ (3 μg/mL) and α-IL-4 (3 μg/mL). T cell depleted splenocytes were added as antigen presenting cells (APC) in the cultures. T_cons_ were derived from congenic CD45.1 mice, and T_regs_ were derived from CD45.2 B6 Foxp3^GFP^ reporter mice so that T_regs_ could also be tracked using CD45.2 staining. In some experiments, where α-IL-6 antibody was added, we restimulated day-1 Th17 cells under Th17 inducing conditions without adding exogenous IL-6. For “Th0” conditions, cells were stimulated with α-CD3, α-CD28, APC and IL-2 (10 ng/mL). For flow cytometry analyses of IL-17A staining, we used unstained controls and stained Th0 cells as negative controls, to define the gates and mark IL-17A+ cells.

### 4.5. Quantitative PCR (q-PCR) Analyses

To measure the relative mRNA levels of TLR-2, Foxp3, ROR-γt and IL-17A, RNA was recovered from T_regs_ or T_cons_ using EZ-10 RNA isolation kit (BioBasic). Genomic DNA was removed by DNA Away (Ambion; LifeTechnologies (AM1906)) and cDNA was synthesized from using MuLV reverse transcriptase enzyme (BioBasic) and OligodT primers. qPCR reaction was performed using SYBR Green PCR Kit (BioBasic) in a real time PCR machine (BioRad). The relative levels were normalized to the amount of β-actin cDNA levels. The following primer sequences were used: β-actin: 5'-AGCCATGTACGTAGCCATCC-3', 5'-CTCTCAGCTGTGGTGGTGAA-3', TLR-2: 5'-CCGGAATTATCAGTCCCAAA-3', 5'-ATCTCCAGCAGGAAAGCAGA-3', ROR-γt: 5'-AACAGGAACAAGTGGCCAAG-3', 5'-GGTAGCTGCCCATCTGAGAG-3', FoxP3: 5'-TTCATGCATCAGCTCTCCAC-3', 5'-TGATCATGGCTGGGTTGTC-3', IL-17A: 5'-AAAGCTCAGCGTGTCCAAA-3', 5'-GCGCCAAGGGAGTTAAAGAC-3'.

### 4.6. C. albicans Infection in Mice

Age and sex matched mice were infected and individually caged after infection, as previously described [22,48]. They were infected under anesthesia by placing a 3 mm diameter cotton ball saturated with PBS (Sham) or 2 × 10^7^ *C. albicans* (CAF2-1) blastospores sublingually for 90 min. Spleen, ALN, CLN, and MOIL were harvested, one or two days after infection for phorbol myrisate acetate (PMA), ionomycin and brefeldin-A re-stimulation, and intracellular cytokine analyses.

### 4.7. IBD Induction by Th17 Cell Transfer *in Vivo*

For Th17 IBD, CD45.1 *Rag1*^−/−^ mice received 8 × 10^5^ CD45.1 Th17 cells that were stimulated and differentiated for 5 days as above. Two weeks later, the mice received fresh CD45.2 T_regs_ derived from congenic CD45.2 Foxp3^GFP^ reporter mice. Spleen, MLN and the gut LP cells were harvested 5 days after T_reg_ injection, for PMA/ionomycin/brefeldin-A re-stimulation and intracellular cytokine analyses.

### 4.8. Statistical Analyses

p values were calculated by Students “t” test in Microsoft Excel software using two tailed distribution and two-sample equal variance parameters, with ** being *p* < 0.05. We also used Mann-Whitney tests, or 2 way ANOVA Sidak’s multiple comparisons test (alpha value 0.05; 95% CI) in Prism 6.0 (GraphPad Software, Inc.) to calculate significance.

## 5. Conclusions

CD4^+^Foxp3^+^T_regs_ produce the effector cytokine IL-17A *in vitro* and *in vivo* during oropharyngeal candidiasis (OPC) in a TLR-2/Myd88 signaling dependent manner. We call these cells as T_reg_-17 cells. Although TLR-2/Myd88 signaling can directly induce proliferation of T_reg_-17 cells, maximal IL-17A induction in T_regs_ requires IL-6, and TLR-2 signaling in APC as well. The minimal and transient loss of Foxp3 expression and suppressive properties are due to the presence of IL-6 in the milieu, but not the direct effect of TLR-2 signaling in T_regs_. Taken together, our data reveal that TLR-2 signaling promotes not only proliferation, but also IL-17A in T_regs_, depending on the cytokine milieu. These T_reg_-17 cells may be relevant in mucosal infections and inflammation.

## Supplementary Materials

Supplementary materials can be accessed at: http://www.mdpi.com/2076-0817/4/1/90/s1.
