# Supplementary material for: TLR-2 Signaling Promotes IL-17A Production in CD4+CD25+Foxp3+ Regulatory Cells during Oropharyngeal Candidiasis
Source: Pathogens. 2015 Mar 17;4(1):90–110. doi: 10.3390/pathogens4010090 (PMC4384074; doi:10.3390/pathogens4010090)
Supplement: Supplementary file 1 [file pathogens-04-00090-s001.pdf]

Supplementary Materials

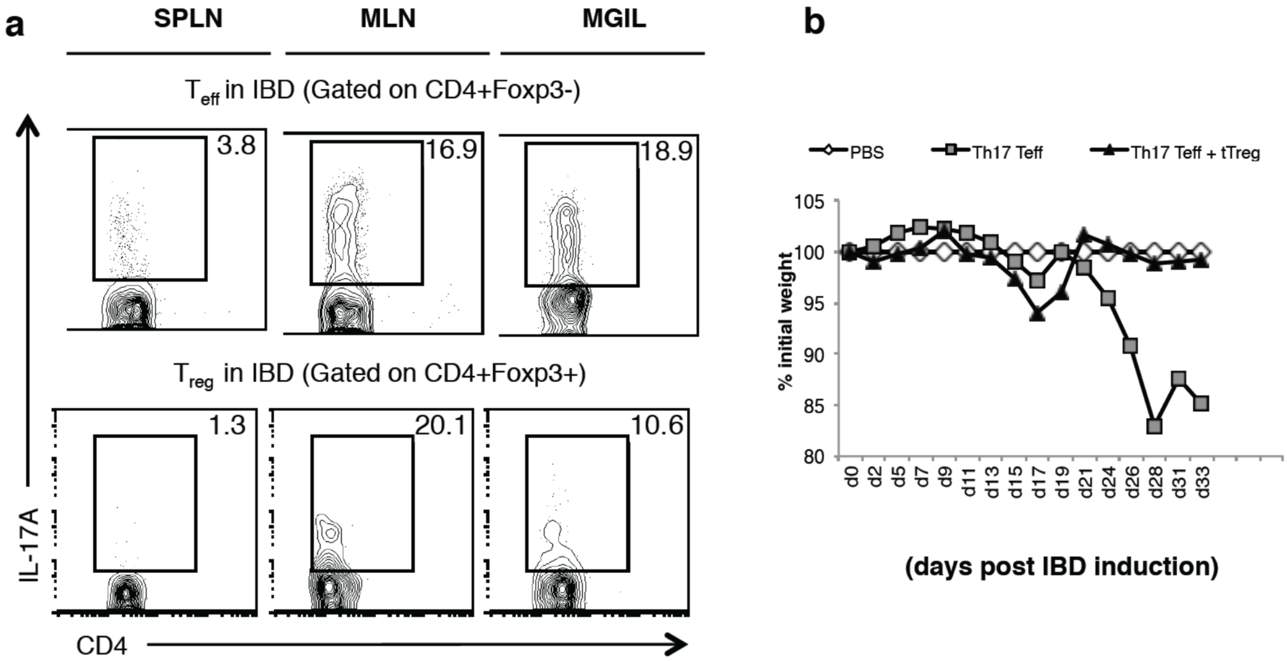

Figure S1. CD4+CD25+Foxp3+ Tregs express IL-17A during IBD *in vivo.* **(A)** IBD was induced in CD45.1 Rag1-/- mice using CD45.1 Th17 Teff colitogenic cells. Two weeks later, to examine the production of IL-17A by Tregs *in vivo*, we injected fresh CD4+ Foxp3GFP+ CD45.2 Tregs isolated from congenic CD45.2 Foxp3GFP transgenic mice. Spleen, mesenteric lymph nodes (MLN) and the mouse gut intraepithelial lamina propria leukocytes (MGIL) were isolated 5 days after Treg injection for intracellular cytokine analyses. Flow cytometric contour plots showing IL-17A expression, gated on CD4+CD45.1+ Foxp3-cells (Teff), or CD4+ CD45.1- Foxp3+ (Treg) cells. **(B)** Treg cells modulate IBD weight loss although a fraction of the cells express IL-17A. IBD was induced by injecting Th17 Teff colitogenic cells (Th17 Teff) (n=10). Control mice received PBS (n=5)(PBS). 2 weeks later, some of the IBD induced mice (n = 5) received thymic Tregs (Th17 Teff + tTreg). Body weight of each mouse in each of the three groups (n = 5) was assessed every two days during the course of the disease. Average body weight of the mice within the groups is shown.

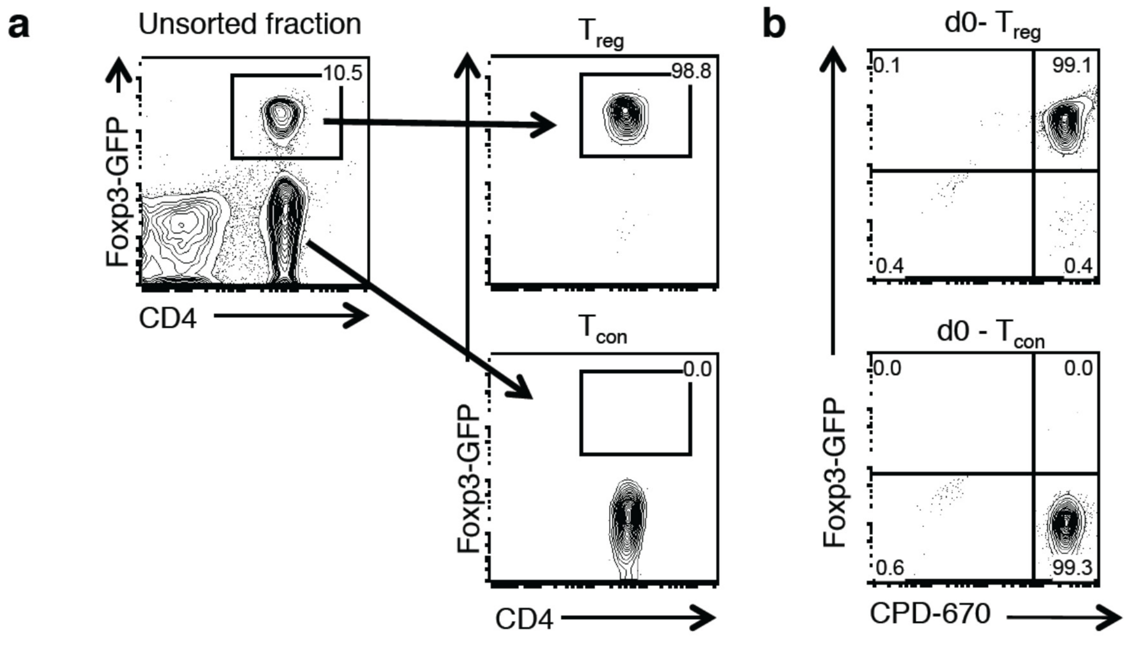

Figure S2. Purity of the isolated cells. **(A)** Flow cytometric contour plots showing CD4 and Foxp3-GFP expression, in Treg or naïve Tcon cells isolated by MACS and FACS from spleen and lymph nodes. (**B**) CPD-670 labeling and Foxp3-GFP expression of the cells on day-0 (d0), isolated *ex vivo* as in "a".

Fig. S3

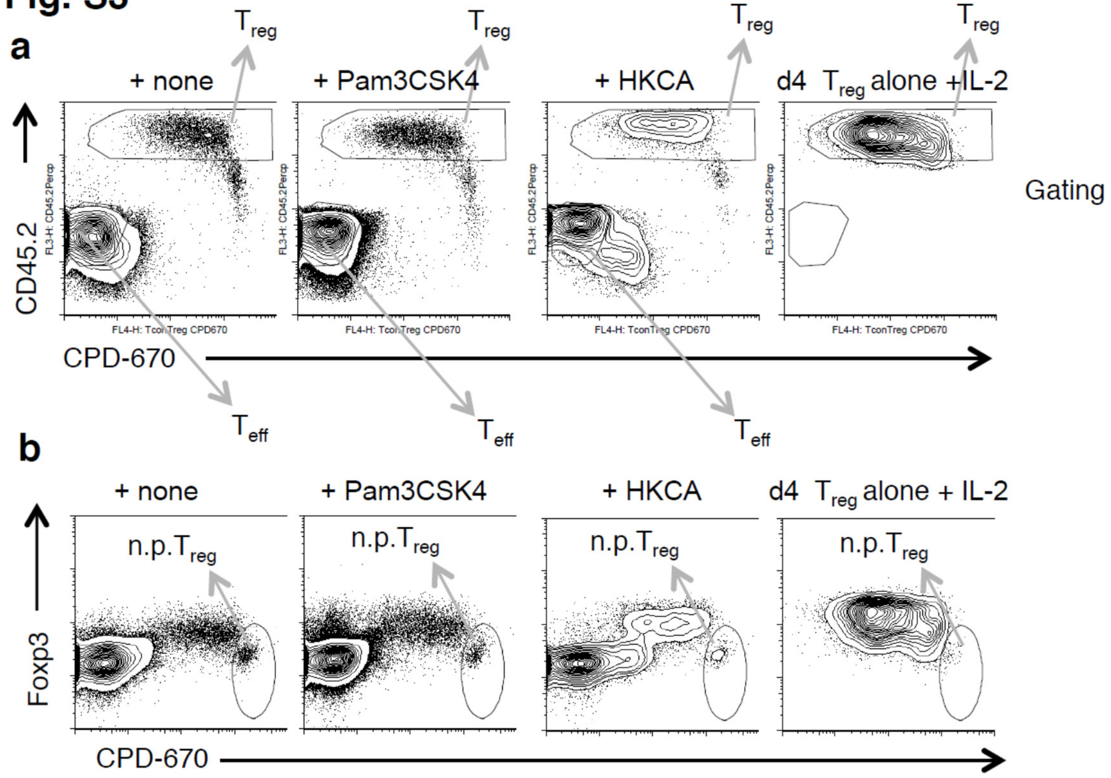

Figure S3. Flow cytometric gating of Treg and Teff cells in co-cultures. Cells were activated in co-cultures as in Fig.4. CD45.2 expression and CPD-670 dilution were assessed by flow cytometry **(A)**. Treg and Teff gates are shown. Foxp3 expression and CPD-670 dilution were assessed by flow cytometry (**B**). Non proliferating (n.p) Tregs that have lost Foxp3 expression are shown in gates.

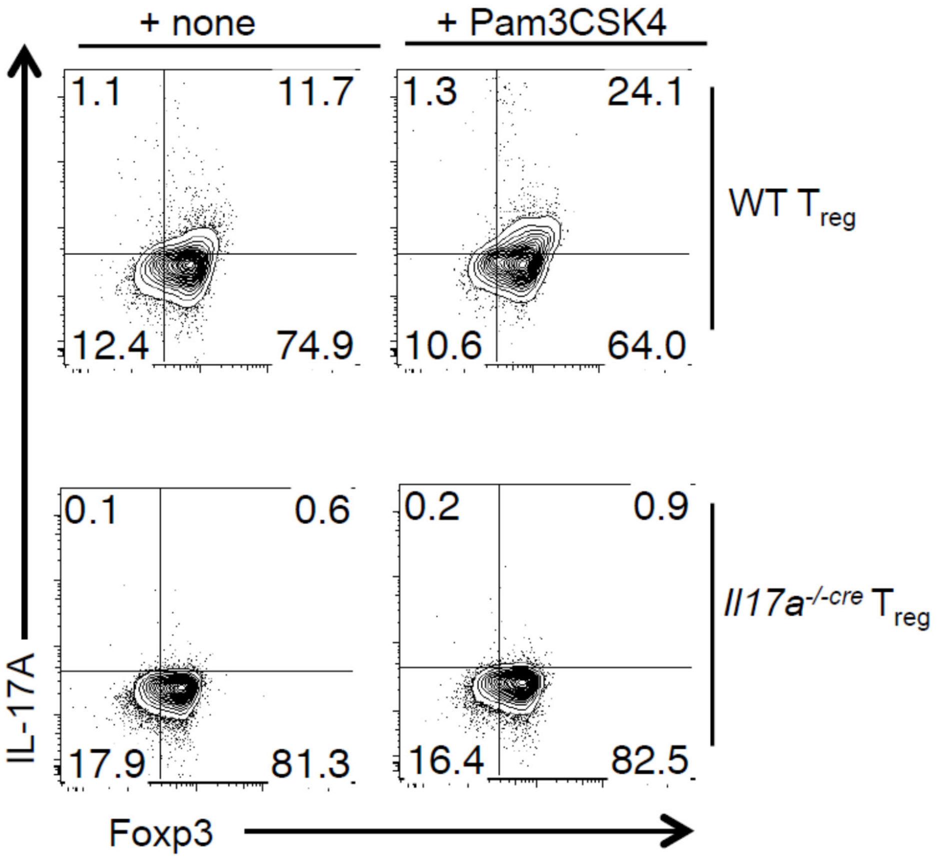

Figure S4. IL-17A staining in IL-17A deficient cells. Cells were activated under Th17 conditions in the presence or absence of Pam3CSK4 for 4 days. Foxp3 expression and IL-17A were assessed by flow cytometry.
